# Prevalence of Ventriculostomy Related Infections and Associated Factors in Low Income Setup

**DOI:** 10.4314/ejhs.v31i6.22

**Published:** 2021-11

**Authors:** Mulualem Wondafrash, Abenezer Tirsit

**Affiliations:** 1 Department of Neurosurgery, St. Paul Hospital Millennium Medical College, Ethiopia; 2 Neurosurgery Division - Department of Surgery, School of Medicine, AAU, Ethiopia

**Keywords:** Ventricuostomy infection, EVD infection, EVD related mortality

## Abstract

**Background:**

Ventriculostomy, a lifesaving and emergency procedure, is used to address raised intracranial pressure. In resource limited set-up like Tikur Annbessa Specialized Referral Hospital, properly designed closed system for ventriculostomy is not available; a device made with sterile pediatric nasogastric tube connected to urine bag is used.

**Methods:**

Institutional based retrospective cross-sectional study conducted on 93 patients with ventriculostomy from January 1, 2009 to June 30, 2018. Ventriculostomy related infection risk described in descriptive statistics and Binary Logistic Regression analysis.

**Results:**

The prevalence rate of Ventriculostomy related infection is 25.8% at Tikur Annbessa Specialized Referral Hospital. Identified risk factors: Ventriculostomy stay for five or more days (AOR=7.676, 95% CI: 1.424, 41.367) and cerbro-spinal fluid leak (AOR=4.592, 95% CI: 1.279, 16.488). Ventriculostomy manipulation showed association on bivariate analysis. K.Pneumoniae (34.6%) and Acinetobacter spp. (30.8%) identified as the main organisms. They were sensitive to combined ceftazidime and vancomycin in 19.2% and meropenem in 42.3%. Based on Tängdén's criteria, 11.8% of patients died of Ventriculostomy related infection while 43% of the patients died in total. Mortality from Ventriculostomy related infection is 45.8% once diagnosed.

**Conclusion:**

The prevalence rate of Ventriculostomy related infection is 25.8% at Tikur Annbessa Specialized Referral Hospital. The duration of external ventricular drain and Cerebrospinal fluid leak are identified risk factors. Ventriculostomy related infection is responsible for one third of mortality related with external ventricular drain. The remaining two third exact causes are not known.

## Introduction

Cerebral ventriculostomy is life-saving procedure. It is frequently used to treat acute hydrocephalus due to intracranial hemorrhage, traumatic brain injury, infection and tumor. It is widely used because the installation technique is quite simple, the cost is affordable for most health systems, and it represents the best option to monitor intracranial pressure (ICP). In addition, external ventricular drain (EVD) allows for cerebrospinal fluid drainage, aiding in the control of intracranial hypertension (1).

Enthusiasm for the use of EVDs has always been tampered somewhat by the increased risk of ventriculomeningitis associated with this modality of ICP monitoring (2). The probability of such risk commonly ranges between 10 to 17 percent. However, it is important to note incidents of 0 to 45 percent have been reported in some cases (3, 4). The factors that predispose a patient to a ventricular catheter infection have been the subject of much research.

A large number of studies focused on causes of infection after ventriculostomy; however, a common cause has not been identified. Several factors have proven to cause Ventriculostomy related infection (VRI). The most reported factor is the amount of time that the draining device remains in the patient. Other contributing factors include the frequency with which the device is changed, the length of subcutaneous device trajectory, associated hemorrhage, skull fracture with CSF leak, and systemic infections (1). Rest of the identified risk factors, such as need for craniotomy and systemic sepsis, are inherent to the patient Population and complicate the relationship between ventricular catheters and meningitis (3). A solid knowledge of the rates and risk factors associated with EVD-related infections would be useful in terms of implementing measures to prevent and control it, since it may lead to worse outcome, including increased mortality, prolonged hospital stay, repeat surgery and increased hospital costs. (5, 6, 7, 8). Using the key word ventriculostomy infection in sub-Saharan Africa we could not find publications in PubMed and Google scholar. Therefore, in this study, we aimed to evaluate patients who required ventriculostomy retrospectively and find out retrospectively and find out the prevalence of ventriculostomy related infection, common associated factors, microbial pattern and mortality in Tikur Annbessa Specialized Referral Hospital (TASH) which is one of the sub-Saharan Hospital in the hope this research will be a benchmark for future researches in this area.

## Methods

The study was conducted at TASH which is a specialized teaching referral hospital located in the Ethiopia's capital, Addis Ababa. It is one of the three governmental hospitals providing neurosurgical services and accepts referrals from all over the country. Basically, in a typical month there will be 2000 outpatient neurosurgical visits and at least 100 neurosurgical procedures per month.

The EVD insertion procedure documented on the chart consists of the following steps. First patient's head is shaved at surgical site; scalp is prepped with povidone iodine solution and draped under aseptic technique. A small incision is made over Kocher's, Frazier's, Dandy's or Keen's point. Then burrhole is done and dura is cauterized and opened, a small cortisectomy is done. Brain cannula is introduced to the ventricle and CSF is taken for analysis. A No. 6/8 sterile pediatric feeding tube is introduced after it had been tunneled from site on scalp approximately 5 cm from initial incision. After the ventricular catheter is inserted into appropriate lateral ventricle functionality is checked by free flow. The catheter is fixed in place with stitch to prevent dislodgment. Then the catheter is connected to sterile urine bag with connector. The connector is covered by iodine-soaked gauze and plastered. Finally, the urine bag is fixed at appropriate height desired to parallel the ICP.

Once ethical clearance was gained all neurosurgical patients who underwent cranial surgery were screened from Operation Theater, laboratory logbook and outpatient log books to look for patients for whom EVD insertion was done from Jan 1, 2009 to Jun 30, 2018. All patient's charts and laboratory results of patients who underwent EVD insertion between Jan 1, 2009 to Jun 30, 2018 were reviewed retrospectively using a standardized questionnaire at the mentioned hospital. From these all patients for whom EVD insertion done for any reason including patient for whom insertion done before major surgery as temporizing measure, during emergency hours, patient who had EVD insertion done during major surgery as prophylaxis or patient for whom insertion done for post op deterioration were included.

Patients were excluded if missing medical records, if patient had meningitis or ventriculitis before the insertion of a catheter, compound depressed skull fracture or basal skull fracture with CSF leak. After all this, the following operational definitions were applied.

**The following operational definitions ere used.**

1. **Ventriculostomy Related Infection**- when fever (>=T_0_ 38.5°C, with/without clinical signs of meningitis) associated with at least one positive CSF culture and at least one element of CSF abnormality, including low CSF glucose levels (<40 mg/dl), high CSF protein (>50 mg/dl), or CSF pleocytosis (>100/mm^3^). (9)

2. **Colonization** is considered to be present when several CSF cultures were positive without CSF abnormality. (9)

3. **Contamination** is defined as an isolated positive CSF culture without CSF abnormality. (9)

4. **Dexamethasone**- if patient has taken dexamethasone for 2 weeks both preop and post op together. (10,11)

5. **Systemic illness/ infection** - any medical illness including diabetes, hypertension or infections other than VRI (according to the Centers for Disease Control and Prevention criteria) (9,12)

6. **Ventriculostomy manipulation**- if the patient had more than one CSF sample taken excluding the one taken during ventriculostomy insertion or if LP was done. Or irrigation of ventricular system was done. (13)

**7. Prophylactic antibiotic-** Antibiotics administrated by the anesthesiologist at induction in the operating room and for 24 hours after the procedure. (5)

8. **Death was considered not to be attributable to VRI /Tängdén criteria/**

If all of the following criteria were met:

2 negative CSF culture results before death (if performed),

Resolving inflammatory parameters,

Resolution of clinical signs of meningitis, and a cause other than VRI was found to be more probable according to the treating physician (14)

The collected data from a prepared questionnaire was coded, cleaned and transported to SPSS version 23.0 software for analysis. Participants' socio-demographic characteristics and other variables are presented using the relevant descriptive statistics. Bivariate analysis was done at 25% level of significance to screen out potentially significant independent variables. Multiple Logistic Regression was performed using the significant independent variables. The association between the dependent variable and independent variables were analyzed using Binary Logistic Regression. The adequacy of the final model was checked using the Hosmer and Lemeshow goodness of fit test and the final model was fitted for the data well (p-value = 0.899). For Binary Logistic Regression, 95% confidence interval was calculated and variables with p-value ≤ 0.05 were considered as statistically significant.

## Results

Out of the 150 patients for whom EVD was done the study was conducted on 93 patients who fulfilled the inclusion criteria. They were selected based on a retrospective review of medical card, laboratory logbook, Operation Theater log book and OPD log book from January 1, 2009 to June 30, 2018. The Sociodemographic data collected were gender, age and residential region of the patients. Over half of study participants (55%) were males while the rest (45%) were female. Further, a significant number of patients (75%) were under the age of 31 years old. Thirty two percent of the study participants were under or equal to 10 years old. The details of socio-demographic characteristics of the study participants are presented in Table 1.

**Table 1:** Socio-demographic characteristics of study participants in Tikur Annbesa specialized referral Hospital, from January 1, 2009 to Jun 30, 2018 (n=93)

Variables	Frequency	Percent (%)
**Sex**		
Male	51	54.8
Female	42	45.2
**Age in years**		
≤10	30	32.3
11–20	17	18.3
21–30	23	24.7
31–40	7	7.5
41–50	4	4.3
51–60	6	6.5
>60	6	6.5
**Address**		
Oromia	28	30.1
Addis Ababa	25	26.9
Amhara	16	17.2
SNNP	13	14.0
Benishangul Gumz	2	2.2
Unknown	9	9.7

**Figure 1:**
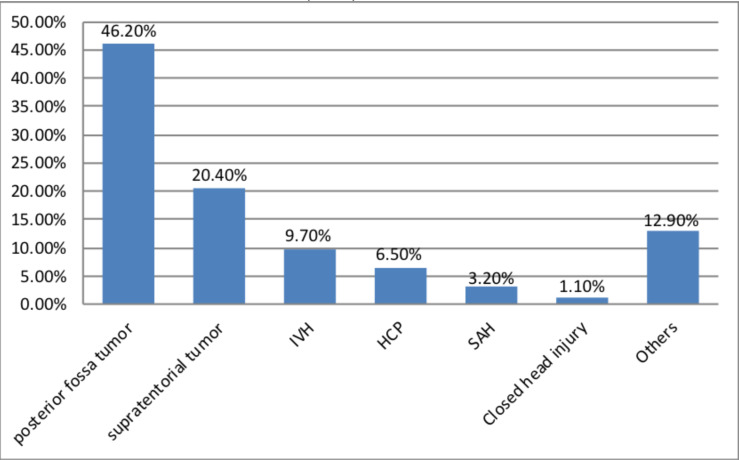
Indications for ventriculostomy among study participants in Black Lion Hospital, from Jan 1, 2009 to June 30, 2018 (n=93).

Among all indications for ventriculostomy infection, posterior fossa tumors account for 46.2% (n=43) followed by supra-tentorial tumors which accounts 20.4% (n=19). In addition, other indications include: Cerebellar stroke, Cerebellar hemorrhage, post op HCP.... that account 12.9% (n=12) (Figure 1).

Looking for Ventriculostomy related infection evidence with clinical and laboratory parameters: Among all study participants, 41 (44.1%) of them had fever of 38.5 °C or more and 31 (33.3%) of the patients had reduced level of consciousness or seizure. Twelve (12.9%) of the patients had low CSF glucose level (<40mg/dl or <50% of serum glucose) and 19 (20.4%) of the patients had high CSF protein (>50mg/dl). Furthermore, 30 (32.3%) of the patients had CSF pleocytosis (>100/mm^3^).

**Figure 2:**
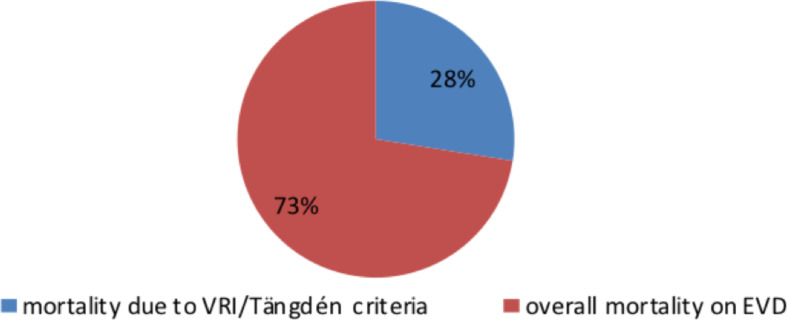
Mortality of patients on ventriculostomy comparing VRI mortality according to Tängdén criteria with the overall mortality.

Among all patients participated in this study, only one patient fulfilled the criteria for contamination and also only one patient fulfilled the criteria for ventriculostomy colonization. Twenty-four (25.8%) patients fulfilled the criteria for ventriculostomy related infection. Eleven (11.8%) patients died of ventriculostomy related infection according to Tängdén criteria and a total of 40 (43%) of the patients died. The details are presented in Table 2 and Figure 2.

**Table 2:** Categorization of patients and short-term status of patients in Tikur Annbessa specialized referral Hospital, from Jan 1, 2009 to Jun 30, 2018 (n=93)

Variables	Frequency	Percent (%)
**Patient fulfill criteria for contamination**		
Yes	1	1.1
No	92	98.9
**Patient fulfill criteria for ventriculostomy** **colonization**		
Yes	1	1.1
No	92	98.9
**Patient fulfill criteria for ventriculostomy related** **infection**		
Yes	24	25.8
No	69	74.2
**Patient died of ventriculostomy related infection** **according to Tängdén criteria**		
Yes	11	11.8
No	82	88.2
**Patient died**		
Yes	40	43.0
No	53	57.0

For the majority (68.8%) of the patients EVD stayed for five or more days. EVD was manipulated on 37 (39.8%) of the patients. Twenty (21.5%) of the patients had post operation CSF leak.

Among the total patients included in this study, 24 (25.8%: 95% CI: 17.2, 35.5) of them developed ventriculostomy related infection and the remaining 69 (74.2%: 95% CI: 64.5, 82.8) of them did not develop ventriculostomy related infection.

At 25% level of significance bivariate analysis; EVD stay for five or more days, post operation CSF leak and EVD manipulation were significantly associated with ventriculostomy related infection. However, in this research only EVD stay for five or more days and post operation CSF leak were significantly associated with ventriculostomy related infection in the multivariable analysis of Binary Logistic regression model at 5% level of significance (Table 3).

**Table 3:** Bivariate and multivariable analysis for factors associated with ventriculostomy related infection in Tikur Annbessa specialized referral Hospital, from Jan1, 2009 to Jun 30, 2018 (n=93)

Variables	Infection		COR (95% CI)	AOR (95% CI)	p-value
				
	Yes	No			
**EVD stay for five or** **more days**					
Yes	21	43	4.233 (1.149, 15.593)	**7.676 (1.424, 41.367)**	**0.018** *
No	3	26	1.00	1.00	
**EVD manipulation**					
Yes	13	24	2.216 (0.863, 5.692)	0.901 (0.276, 2.941)	0.862
No	11	45	1.00	1.00	
**Post op CSF leak**					
Yes	20	3	3.164 (1.109, 9.023)	**4.592 (1.279, 16.488)**	**0.019** *
No	12	5	1.00	1.00	

**Note**: COR, Crude odds ratio; AOR, Adjusted odds ratio; CI, Confidence interval

* Statistically significant factors in multivariable analysis

According to CSF Grams' stain of patients on ventriculostomy, no bacteria were seen on 74 (79.6%) of the patients and bacterial growth on CSF culture was seen on only 26 (29%) cases. Among those who had bacterial growth on CSF culture, 20 (76.9%) of them were culture positive once.

The main organisms were *Klebsiellapneumoniae* and *Acinetobacter*. The cultured organisms were sensitive for combined ceftazidime and vancomycin on five (19.2%) cases. While for Meropenem eleven (42.3%) cases were sensitive. In addition, the cultured organisms were sensitive for Amikacin on 14 (53.8%) cases. In eleven (42.3%) cases they were sensitive to one of the following: Tobramycine, Chloramphenicol, Erytromycin, Clindamycin, cloxacillin, Cefoxitin, Doxycycline, Tetracycline and polymyxin. The details are presented in Table 4.

**Table 4:** Microbial studies among patients on ventriculostomy in Tikur Annbessa specialized referral Hospital, from Jan 1, 2009 to Jun 30, 2018

Variables	Frequency	Percent (%)
**CSF Gram's stain (n=93)**		
Gram positive	2	2.2
Gram negative	9	9.7
No Bacteria seen	74	79.6
Other aspect (not done)	8	8.6
**Organism growth on CSF culture (n=93)**		
Yes	26	29.0
No	67	71.0
**How many times culture positive (n=26)**		
Once	20	76.9
twice	4	15.4
three times	2	7.7
**List of organisms (n=26)**		
K. Pneumoniae	9	34.6
Acinetobacter	8	30.8
Pseudomonas	2	7.7
E.Coli	1	3.8
Enterobacter and Acinetobacter	1	3.8
K. Pneumoniae and SerratiaSpp	1	3.8
K. Pneumoniae and Acinetobacter	1	3.8
K. Oxytoca	1	3.8
Staphylococcus Aureus	1	3.8
Viridans Streptococcus	1	3.8
**The organism/ organisms sensitive for** **ceftazidime + vancomycin (n=26)**		
Yes	5	19.2
No	21	80.8
**The organism/ organisms sensitive for** **meropenem (n=26)**		
Yes	11	42.3
No	15	57.7
**Sensitive for other drugs (n=26)**		
Amikacin	14	53.8
Amoxicillin/clavulanate	1	3.8
Othersdrugs	11	42.3

Others drugs Include: Tobramycine, Choramphenicol, Erythromycin, Clindamycin, cloxacillin, Cefoxitin, Doxycycline, Tetracycline and polymyxin

## Discussion

In the 93 patients enrolled in this research, we did not find a significant relationship between socio-demographic data and ventriculostomy related infections. Although one third of study participants were under 10 years of age, the ratio of male to female is comparable.

The prevalence rate of VRI is 25.8% at TASH, which could be similar to other resource limited set-ups. When we compared this with other reports from high income countries that use standard equipment, we found out prevalence as high as 45 % and to our surprise there was no significant difference observed from the average rate as well which lies 17% (3,10,11). When one considers the limitation of material resources, health professional, set up of patient care and patient overload in low income set up of TASH this may be fair even though there is still room for further improvement.

The overall mortality of patients on ventriculostomy is 43%. While 11.8% mortalities where specifically attributable from ventriculostomy related infection according to Tängdén criteria. The remaining two third exact cause is not known. This means VRI mortality accounts for only one third of deaths of patients while on ventriculostomy unlike the assumption previously. There are other factors in play that need to be addressed by future research. From our daily practice we can consider patients not strictly followed by trained professional familiar with EVD, attendant's knowledge, no guideline for caring a patient on EVD and patient on EVD being treated in the ward. When we see the mortality of patients once ventriculostomy related infection is diagnosed it becomes almost fifty percent (45.8%). This is in contrary to VRI not having effect on overall mortality (9).

According to multivariate analysis of binary logistic regression, patients with EVD stay for five or more days were 7.676 times more likely to develop ventriculostomy related infection compared to those patients with shorter EVD stay (AOR=7.676, 95% CI: 1.424, 41.367). Patients who had post-operative CSF leak were 4.592 times more likely to develop ventriculostomy related infection compared to those patients who had no post operation CSF leak (AOR=4.592, 95% CI: 1.279, 16.488). These findings are in line with other previous researches (1,5, 15, 16, 17, 18, 19, and 20).

Although EVD manipulation did not show significant correlation on multi variate analysis in this study, it had significance on bivariate analysis. This result implies we should consider this in our day-to-day clinical practice even though statically not significant. However, literatures show there is increased risk with manipulation of EVD (13,20).

Further, the study finding indicated that posterior fossa tumors are predominant indication for Ventriculostomy. In fact, it contributes to 46% for ventriculostomy indication. This could be due to TASH being the only governmental center where these tumors are operated. No correlation was found between reason for insertion and VRI in this study. This finding contradicts with finding of increased risk with subarachnoid hemorrhage and intraventricular hemorrhage in other researches (2, 9,13).

Even though conflicting results are there regarding dexamethasone use and VRI, this study shows no effect of dexamethasone use more than two weeks. This goes with Holloway et al findings (3,10,11). Also, the absence of VRI relation with medical history of systemic illness/ infection in this research correlates with findings in the Brazilian study (1).

Given there were heterogeneous and suboptimal available data regarding the issue of prophylactic antibiotic use, this research attempted to find similar or contrary evidence in TASH. The result suggested that giving prophylactic antibiotics on induction or post op for 24 hours has no correlation with VRI. This finding is parallel with international literatures (15, 17, 21).

Further changing Ventriculostomy has no effect on risk of infection as in Lozier's finding (3, 2). This is in contradiction with the result of other researches which showed increasing risk of infection with increasing number of EVD insertion(7, 10). The finding of lack of relation between VRI and neurosurgical procedure, other than ventriculostomy done at same operation, also contradict with what other literatures published(2, 9).

According to the microbial pattern finding of the study, *Klebsiella pneumoniae* (35%) and *Acinetobacter spp*. (31%) were the two organisms identified. These results are similar with the finding that gram negatives being predominant cause of VRI. In addition, 58% mortality in Gram's negative VRI in the literatures is comparable to the finding of 50% mortality in this research (16, 20, 22,23). Still, this is contrary to finding of gram positives accounting to more than two third of nosocomial CSF infections (24, 25, 26, 27).

In general, Culture positive organisms were sensitive to the empiric combined therapy of ceftazidime and vancomycin only in 19.2 % (24, 28). While for meropenem sensitivity was 42.3%. These findings parallel with the development of microbial resistance in Africa (29). On the other hand, the organisms were sensitive to Amikacin in 53.8% of the patients.

The retrospective nature of this study results in various limitations such as loss of values and selection bias. In order to limit these effects on the study, exclusion criteria where developed and strictly followed. However, this resulted in small number of sample size. Despite these limitations, the researcher believes this study provides credible contributions as it is the first of its kind in Sub Saharan Africa.

Based on the findings, the researcher recommends limiting duration of EVD stay as much as possible either via early removal of EVD or shunting patients. Improved follow up after insertion of ventriculostomy including placing of the ventriculostomy at appropriate height, adequate wound closure and care to prevent CSF leak. Moreover, proper follow up and early identification and treatment of CSF leak is of paramount importance.

Furthermore, amikacin should be considered as part of empiric treatment for VRI, and giving a combination of ceftazidime and vacomycin as empiric treatment should be questioned although a large prospective study is needed.
